# Variations in symptoms, endoscopy use and emergency diagnosis of colorectal cancer by body mass index: a retrospective cohort study using linked electronic health records in England

**DOI:** 10.1136/bmjopen-2025-107468

**Published:** 2026-05-07

**Authors:** Jessica Sarah Kurland, Aradhna Kaushal, Sara Benitez Majano, Georgios Lyratzopoulos, Cristina Renzi

**Affiliations:** 1 UCL Institute of Epidemiology and Health Care, London, UK; 2 Inequalities in Cancer Outcomes Network (ICON) Group, Department of Non-Communicable Disease Epidemiology, London School of Hygiene & Tropical Medicine, London, UK; 3 Epidemiology of Cancer Healthcare & Outcomes (ECHO) Research Group, Department of Behavioural Science and Health, UCL Institute of Epidemiology and Health Care, London, UK; 4 Faculty of Medicine, Università Vita Salute San Raffaele, Milan, Italy

**Keywords:** Clinical Decision-Making, Obesity, Epidemiology, Gastrointestinal tumours, Early Detection of Cancer

## Abstract

**Abstract:**

**Objectives:**

Body mass index (BMI) confers a higher risk of colorectal cancer (CRC) and may influence cancer diagnostic pathways. We investigated variations in diagnostic pathways by BMI category among patients with symptomatic CRC.

**Design:**

Retrospective cohort study using linked cancer registry, primary, and secondary care data.

**Setting:**

England

**Participants:**

5571 patients with symptomatic CRC diagnosed in England between 2011 and 2015.

**Primary and secondary outcome measures:**

Route to CRC diagnosis (emergency presentation and fast-track referrals among patients with new-onset red flag symptoms), presenting symptoms and pre-diagnostic endoscopy use.

**Results:**

Red-flag symptoms (change in bowel habit, rectal bleeding) were more frequently recorded among patients with rectal cancer with obesity and overweight versus normal weight (65.2% and 65.5% vs 56.8%, respectively). Among colon cancer patients endoscopy during the year pre-diagnosis was used in a greater proportion of patients with obesity versus normal weight (72.8% vs 64.4%, p<0.001). Among patients with colon cancer with red-flag symptoms, being overweight versus normal weight was associated with higher odds of fast-track referral compared with diagnosis through other routes (OR: 1.48, 95% CI 1.16 to 1.88). Obesity was associated with lower odds of emergency presentation, compared with normal weight (colon 23.6% vs 32.1%; adjusted OR: 0.72, 95% CI 0.57 to 0.90; rectum 8.3% vs 14.8%; OR: 0.57, 95% CI 0.35 to 0.92).

**Conclusions:**

Patients with CRC with higher BMI are more likely to be referred urgently and less likely to experience emergency cancer diagnosis than normal weight patients.

**STRENGTHS AND LIMITATIONS OF THIS STUDY**

- A population-based cohort study analysing high-quality linked primary care, secondary care and cancer registry data.
- Body mass index (BMI) was based on clinically recorded measurements pre-cancer diagnosis.
- Exclusion of patients with no available BMI data introduces potential bias.

## Introduction

 Improving early diagnosis of cancer and reducing emergency presentations (EPs) are key public health priorities.^1 2^ Compared with non-emergency routes, EPs are associated with later stage at diagnosis, worse survival independently of stage,^3^,^7^ worse patient experiences^8 9^ and increased pressure on healthcare systems.^10^ Nearly one in four colorectal cancers (CRC) in England are diagnosed following EP.^11^ While some EPs are unavoidable, many patients with CRC present to primary care with relevant symptoms prior to EP,^12^ representing potential missed opportunities for earlier diagnosis. A preferential route to diagnosis for patients with symptomatic cancer is through fast-track referrals for diagnostic investigations (also known as Two Week Wait referrals), which should be used when general practitioners (GPs) have suspicion of cancer.

Investigating the relationship between sociodemographic and clinical characteristics, and route to cancer diagnosis reflects an important and emerging area.^13^ Body mass index (BMI) represents a particularly important characteristic to consider given the high prevalence of obesity in the UK^14^ and its role as a risk factor for CRC development^15^,^18^ and prognosis^19^,^21^; however, limited evidence is available. One study investigated the association between BMI and EP of CRC in Canada,^22^ finding EP rates were highest among those with class III obesity (BMI>40 kg/m^2^) and underweight (BMI<25 kg/m^2^) compared with overweight (BMI 25–29 kg/m^2^) or obesity (BMI 30–39 kg/m^2^), while a second looked at body composition and colon cancer EP in Scotland^23^ and found low BMI was associated with increased stage at diagnosis and EP. Four studies found no association between obesity and stage at diagnosis for a variety of cancers in Scotland,^23^ the USA^24 25^ and New Zealand.^26^ Various mechanisms^27^ have been proposed to explain how comorbidities may influence the diagnostic process. These include the alternative explanations mechanism, which may lead to less timely diagnosis if symptoms of an as-yet undiagnosed cancer are attributed to pre-existing conditions, and the surveillance effects, which can reduce the risk of EP if the monitoring of pre-existing comorbidities offers opportunities for early cancer diagnosis^27^ (box 1). Over 90% of colon cancers are diagnosed after symptomatic presentations and they have worse outcomes than screening-detected individuals;^28^ thus, we decided to focus on patients presenting with possible cancer symptoms to primary care. The study aimed to investigate variations in the route to CRC diagnosis and endoscopy use by BMI category among patients with symptomatic CRC.

Box 1 **BMI and Timely cancer diagnosis: Hypothesised mechanisms of Influence**

**Possible mechanisms interfering with timely cancer diagnosis**

*Alternative explanations:* CRC symptoms may be attributed to BMI or other conditions related to BMI instead of CRC. For example, GPs may be more likely to attribute CRC symptoms to benign gastrointestinal conditions, like irritable bowel syndrome and haemorrhoids, which are also associated with higher BMI^37^ in patients with overweight or obesity.

*Stigma and help-seeking behaviour*: engagement in help-seeking behaviour may vary with BMI. Experience of weight stigma may lead to healthcare avoidance for some individuals.^46^,^48^

**Mechanisms facilitating timely cancer diagnosis**

*Surveillance effects*: individuals with a very low or high BMI may have more frequent primary care consultations, either relating directly to weight management or to morbidity associated with weight, providing additional opportunities for diagnosis.

*Risk factors and cancer suspicion*: overweight and obesity are risk factors for CRC.^49^ GP awareness of this may increase CRC suspicion for patients with higher BMI, resulting in more prompt referral for diagnosis.

**Mechanisms facilitating and/or interfering with timely cancer diagnosis**

*Pathological theory*: multiple biological mechanisms have been proposed to explain associations between obesity and CRC^50^ and there is evidence that obesity is associated with differences in CRC tumour characteristics at the metabolic level.^51^ Pathological differences related to BMI could impact cancer aggressiveness or symptomatic presentations, possibly making CRC easier or more difficult to detect.

BMI, body mass index; CRC, colorectal cancer; GP, general practitioner.

## Methods

### Study design and participants

We conducted a retrospective cohort study of patients with symptomatic CRC aged 30–99 diagnosed in England between 2011 and 2015. Participants were identified in the National Cancer Registry Analysis (NCRAS) using International Classification of Disease 10th Edition (ICD-10) codes (colon: C18.1-C18.9, rectal: C19, C20). NCRAS contains information on all cancers in people living in England^29^ and provides information on cancer site, date and tumour, node, metastases stage at diagnosis, age at diagnosis and route to diagnosis. The Clinical Practice Research Datalink (CPRD) and Hospital Episode Statistics (HES)—primary and secondary care databases—provided information on BMI, symptoms, comorbidities and endoscopy use. Linkage between NCRAS, CPRD and HES is carried out by the Trusted Third Party NHS Digital, to a high quality.^30^

**Figure 1:**
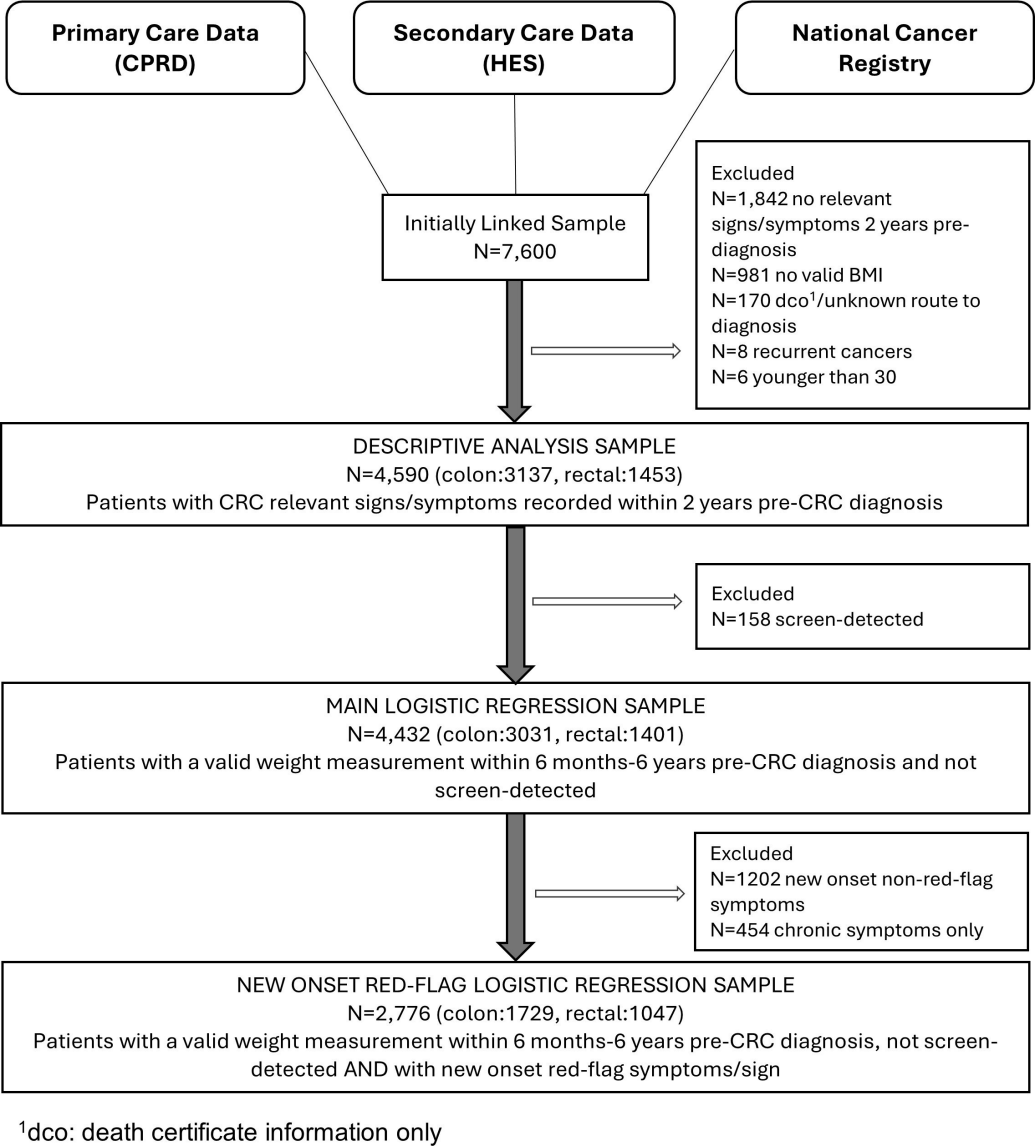
Participant exclusion flow chart. Flow diagram showing the data sources used and the number of participants eligible and excluded at each stage of analysis. BMI, body mass index; CPRD, Clinical Practice Research Datalink; CRC, colorectal cancer; HES, Hospital Episode Statistics.

Similar to our previous publications^31 32^ patients were symptomatic if they had at least one CRC-relevant sign/symptom (online supplemental box S1) recorded in primary care within 2 years pre-diagnosis. Patients were excluded if they had missing data on deprivation or route to diagnosis, or a history of cancer at the same site (figure 1).

### Study variables

Outcome variables included EP and fast-track referral, defined by the Routes to Diagnosis Algorithm^28^ (box 2). EPs are cancer diagnoses following presentation to accident and emergency, an emergency GP referral or through emergency inpatient/outpatient pathways. Fast-track referrals for diagnostic investigations are urgent GP referrals for patients with suspicion of cancer, according to the UK National Institute for Health and Care Excellence (NICE) guidelines.^33^ In line with our previous work,^31^ we also evaluated lower gastrointestinal endoscopy use (colonoscopy or flexible sigmoidoscopy) before the cancer diagnosis and after the first presentation with a relevant symptom recorded in primary care during the 2 years pre-cancer diagnosis.

Box 2 **Routes to Diagnosis based on the Routes to Diagnosis Algorithm^28^**

*Emergency presentation*: diagnosis of cancer following presentation to accident and emergency, and emergency GP referral or through emergency inpatient/outpatient pathways.

*Fast-track referral (Two Week Wait referrals)*: diagnosis of cancer following an urgent referral from a GP for suspected cancer.

*Routine GP referral*: diagnosis of cancer following a referral from a GP but not under the ‘fast-track’ pathway.

*Other outpatient/inpatient: elective outpatient/inpatient*: an elective route starting with an outpatient appointment, either consultant to consultant referral or other referral; or starting with inpatient admission, where no earlier information is available from the waiting list prior to admission.

GP, general practitioner.

The main explanatory variables included weight and height data, extracted from CPRD and coded following previous studies,^34^ using measurements closest to the date of diagnosis within the 6-month to 6-year pre-diagnosis period. Weight measurements recorded in the 6 months pre-diagnosis were excluded to avoid bias, as cancer could result in weight loss (reverse causation). Weight measurements recorded more than 6 years pre-diagnosis were also excluded as BMI can change over time. BMI was categorised as underweight <18.5 kg/m^2^; normal weight 18.5–24.9 kg/m^2^; overweight 25–29.9 kg/m^2^ and obese ≥30 kg/m^2^. Individuals with missing BMI were excluded from the main analyses.

Further explanatory variables were symptoms classified into four categories: new-onset red-flag symptoms according to NICE guidelines (change in bowel habit or rectal bleeding), new-onset iron-deficiency anaemia (a red-flag sign), new-onset non-red-flag symptoms (eg, abdominal pain, diarrhoea, fatigue) and only chronic symptoms. Symptoms were defined as new onset if they were recorded in primary care within 24 months pre-diagnosis and with no previous record of the same symptom during the 3–5 years pre-diagnosis. They were defined as chronic if recorded during both the 24 months and the 3- to 5-year pre-diagnosis period. Symptoms were extracted using previously defined medcode lists.^31^ Furthermore, we calculated the Charlson Comorbidity Index (CCI), which provides a weighted disease burden score based on the presence of 17 chronic conditions (online supplemental box 2).^35^ In the analyses, we also included the number of primary care visits for any reason during the 12 months pre-CRC diagnosis (excluding the 30 days), similar to previous work.^12^ Visits within 30 days pre-diagnosis were excluded as they likely relate to previous diagnostic testing (eg, receiving test results) and would not represent new or missed opportunities for diagnosis.

### Statistical analysis

We used descriptive analyses to examine patient sociodemographic and clinical characteristics by BMI category. To investigate associations between BMI category and EP or fast-track referrals, we used multivariable logistic regression models. Unadjusted, partially adjusted (controlling for sociodemographic factors) and fully adjusted (controlling for sociodemographic factors, comorbidity burden, symptoms, endoscopy use and number of primary care consultations) models were run for each outcome. Three potential interactions with BMI category were considered a priori (sex, age and deprivation) and evaluated using likelihood ratio tests. Analyses examining fast-track referrals as the outcome of interest were restricted to the subgroup of patients with new onset red-flag symptoms or anaemia as they are expected to receive fast-track (Two Week Wait) referrals in the UK according to NICE guidelines.^33^

Analyses were conducted separately for colon and rectal cancer, and robust SEs were used to account for clustering of patients by general practice.

We performed sensitivity analyses using regression models to check if a 3-year versus 6-year cut-off for weight measurements, including a separate category for those with missing BMI, and individual common comorbidities instead of CCI, resulted in different estimates from the main findings. Individual comorbidities (cardiovascular disease (myocardial infarction, congestive heart failure, peripheral vascular disease or cerebrovascular disease), chronic obstructive pulmonary disease and diabetes) were identified using ICD-10 codes recorded within HES during the 6 years prior to and up to CRC diagnosis.^31^

All analyses were carried out using STATA statistical software.

### Patient and public involvement

None.

## Results

Out of 7600 patients with CRC, 5571 (colon n=3766, rectum n=1805) patients had at least one relevant symptom recorded in primary care pre-cancer diagnosis and 4432 (82%) patients had a valid BMI measurement (figure 1). Overweight was the most common category (32%) followed by normal weight (26%), obese (23%) and underweight (1.8%). Sociodemographic characteristics varied between BMI categories, with patients with overweight and obesity being more likely to be male, having higher levels of deprivation and having CCI 3+ comorbidity index, compared with normal weight individuals (table 1).

**Table 1:** Sociodemographic characteristics and comorbidities by BMI category (N=4590)

	**All patients N (column%)**	**Underweight**	**Normal weight**	**Overweight**	**Obese**	**P value** ^*^
**Colon cancer**						
Sex						
Female	1575 (50.21)	56 (77.78)	563 (56.13)	560 (45.98)	396 (46.92)	<0.001
Male	1562 (49.79)	16 (22.22)	440 (43.87)	658 (54.02)	448 (53.08)	
Age						
<45	66 (2.10)	2 (2.78)	26 (2.59)	22 (1.81)	16 (1.90)	<0.001
45–54	195 (6.22)	3 (4.17)	57 (5.68)	61 (5.01)	74 (8.77)	
55–64	424 (13.52)	3 (4.17)	102 (10.17)	158 (12.97)	161 (19.08)	
65–74	795 (25.34)	17 (23.61)	217 (21.64)	314 (25.79)	247 (29.27)	
75–84	1137 (36.24)	28 (38.89)	367 (36.59)	464 (38.10)	278 (32.94)	
85+	520 (16.58)	19 (26.39)	234 (23.33)	199 (16.34)	68 (8.06)	
Deprivation quintile						
1 (lowest)	761 (24.26)	19 (26.39)	275 (27.42)	295 (24.22)	172 (20.38)	0.012
2	699 (22.28)	10 (13.89)	240 (23.93)	267 (21.92)	182 (21.56)	
3	677 (21.58)	16 (22.22)	203 (20.24)	274 (22.50)	184 (21.80)	
4	568 (18.11)	16 (22.22)	169 (16.85)	216 (17.73)	167 (19.79)	
5 (highest)	432 (13.77)	11 (15.28)	116 (11.57)	166 (13.63)	139 (16.47)	
Comorbidity						
CCI						
0	1512 (48.20)	24 (33.33)	473 (47.16)	618 (50.74)	397 (47.04)	0.156
1	763 (24.32)	25 (34.72)	253 (25.22)	279 (22.91)	206 (24.41)	
2	380 (12.11)	12 (16.67)	128 (12.76)	135 (11.08)	105 (12.44)	
3+	482 (15.36)	11 (15.28)	149 (14.86)	186 (15.27)	136 (16.11)	
CVD	651 (20.75)	19 (26.39)	215 (21.44)	274 (22.50)	143 (16.94)	0.0101
COPD	632 (20.15)	30 (41.67)	213 (21.24)	215 (17.65)	174 (20.62)	<0.001
Diabetes	587 (18.71)	5 (6.94)	144 (14.36)	209 (17.16)	229 (27.13)	<0.001
Total (% of total)	3137 (100)	72 (1.91)	1003 (26.63)	1218 (32.34)	844 (22.41)	
**Rectal cancer**						
Sex						
Female	574 (39.50)	16 (59.26)	190 (42.51)	199 (35.04)	169 (41.12)	0.0101
Male	879 (60.50)	11 (40.74)	257 (57.49)	369 (64.96)	242 (58.88)	
Age						
<45	38 (2.62)	0 (0.00)	16 (3.58)	13 (2.29)	9 (2.19)	<0.001
45–54	131 (9.02)	2 (7.41)	31 (6.94)	57 (10.04)	41 (9.98)	
55–64	273 (18.79)	3 (11.11)	72 (16.11)	96 (16.90)	102 (24.82)	
65–74	413 (28.42)	4 (14.81)	118 (26.40)	160 (28.17)	131 (31.87)	
75–84	428 (29.46)	8 (29.63)	135 (30.20)	184 (32.39)	101 (24.57)	
85+	170 (11.70)	10 (37.04)	75 (16.78)	58 (10.21)	27 (6.57)	
Deprivation quintile						
1 (lowest)	322 (22.2)	5 (18.52)	103 (23.04)	150 (26.41)	64 (15.57)	0.013
2	309 (21.27)	3 (11.11)	100 (22.37)	117 (20.60)	89 (21.65)	
3	330 (22.71)	8 (29.63)	99 (22.15)	123 (21.65)	100 (24.33)	
4	261 (17.96)	5 (18.52)	78 (17.45)	104 (18.31)	74 (18.00)	
5 (highest)	231 (15.90)	6 (22.22)	67 (14.99)	74 (13.03)	84 (20.44)	
Comorbidity						
CCI						
0	843 (58.02)	6 (22.22)	275 (61.52)	331 (58.27)	231 (56.20)	0.007
1	314 (21.61)	11 (40.74)	80 (17.90)	126 (22.18)	97 (23.60)	
2	136 (9.36)	6 (22.22)	42 (9.40)	55 (9.68)	33 (8.03)	
3+	160 (11.01)	4 (14.81)	50 (11.19)	56 (9.86)	50 (12.17)	
CVD	221 (15.21)	8 (29.63)	72 (16.11)	83 (14.61)	58 (14.11)	0.16
COPD	235 (16.17)	11 (40.74)	75 (16.78)	90 (15.85)	59 (14.36)	0.004
Diabetes	230 (15.83)	3 (11.11)	41 (9.17)	91 (16.02)	95 (23.11)	<0.001
Total (% of total)	1453 (100)	27 (1.50)	447 (24.76)	568 (31.47)	411 (22.77)	

BMI: underweight <18.5 kg/m2, normal weight 18.5–24.9 kg/m2, overweight 25–29.9 kg/m2, obese >=30 kg/m2.

* χ2 p value.

BMI, body mass index; CCI, Charlson Comorbidity Index; COPD, chronic obstructive pulmonary disease; CVD, cardiovascular disease (myocardial infarction, congestive heart failure, peripheral vascular disease or cerebrovascular disease).

### Clinical characteristics by BMI category

Symptoms recorded pre-cancer diagnosis differed by BMI for rectal, but not colon, patients with cancer, with change in bowel habit/rectal bleeding being more common and anaemia less common among patients with overweight and obesity compared with normal and underweight (table 2). The proportion of patients with an endoscopy during the 12 months pre-CRC diagnosis was greater among those with overweight and obesity compared with the normal and underweight groups (table 2). This difference was most obvious for colon cancer, where 72.8% of patients with obesity underwent endoscopic procedures compared with 64.4% of patients with normal weight (p<0.001). There was no difference in the number of primary care consultations between BMI categories, except for the underweight group, who had fewer consultations (online supplemental table 1).

**Table 2:** Symptomatic presentation, lower GI endoscopy use, route to diagnosis and stage at diagnosis by BMI category

	All patients N (column%)	Underweight	Normal weight	Overweight	Obese	P value
**Colon cancer**						
New onset symptomatic presentation						
CIBH/RB	724 (23.07)	12 (16.67)	219 (21.83)	284 (23.32)	209 (24.76)	0.216
Anaemia	1061 (33.83)	21 (19.17)	336 (33.50)	417 (34.24)	287 (34.00)	
Non red-flag	992 (31.62)	34 (47.22)	326 (32.50)	379 (31.1)	253 (29.98)	
Chronic only	360 (11.48)	5 (6.94)	122 (12.16)	138 (11.33)	95 (11.26)	
Had lower GI endoscopy after first CRC relevant symptomatic presentation						
Yes	2117 (67.47)	41 (56.94)	646 (64.41)	816 (67.00)	614 (72.75)	<0.001
No	1020 (32.53)	31 (43.06)	357 (35.59)	402 (33.00)	230 (27.25)	
Route to diagnosis						
Emergency	875 (27.90)	29 (40.28)	322 (32.10)	325 (26.68)	199 (23.58)	<0.001
Fast-track	981 (31.27)	16 (22.22)	286 (28.51)	419 (34.40)	260 (30.81)	
GP referral	801 (25.53)	16 (22.22)	261 (26.02)	292 (23.97)	232 (27.49)	
Other in/out	374 (11.92)	10 (13.89)	110 (10.97)	143 (11.74)	111 (13.15)	
Screening	106 (3.38)	1 (1.39)	24 (2.39)	39 (3.20)	42 (4.98)	
TNM stage at diagnosis†						
I	277 (8.83)	2 (4.08)	86 (11.33)	104 (10.73)	85 (12.52)	0.627^4^
II	781 (24.91)	22 (44.90)	242 (31.88)	305 (31.48)	212 (31.22)	
III	681 (21.71)	14 (28.57)	211 (27.80)	261 (26.93)	195 (28.72)	
IV	717 (22.87)	11 (22.45)	220 (28.99)	299 (30.86)	187 (27.54)	
Missing‡	681 (21.69)	23 (31.94)	244 (24.33)	249 (20.44)	165 (19.55)	
**Rectal cancer**	All patients N (column%)	Underweight	Normal weight	Overweight	Obese	P value^*^
New onset symptomatic presentation						
CIBH/RB	904 (62.22)	10 (37.04)	254 (56.82)	372 (65.49)	268 (65.21)	<0.001
Anaemia	165 (11.36)	6 (22.22)	63 (14.09)	52 (9.15)	44 (10.71)	
Non red-flag	290 (19.96)	6 (22.22)	93 (20.81)	110 (19.37)	81 (19.71)	
Chronic only	144 (9.91)	55 (18.52)	37 (8.28)	34 (5.99)	18 (4.38)	
Had lower GI endoscopy after first CRC relevant symptomatic presentation						
Yes	1240 (85.36)	17 (62.96)	369 (82.55)	493 (86.80)	361 (87.83)	0.001
No	213 (14.66)	10 (37.04)	78 (17.45)	75 (13.20)	50 (12.17)	
Route to diagnosis						
Emergency	167 (11.49)	8 (29.63)	66 (14.77)	59 (10.39)	34 (8.27)	0.005
Fast-track	654 (45.00)	7 (25.93)	192 (42.95)	254 (44.72)	201 (48.91)	
GP referral	435 (29.94)	7 (25.93)	122 (27.29)	187 (32.92)	119 (28.95)	
Other in/out	145 (9.98)	4 (14.81)	47 (10.51)	55 (9.68)	39 (9.49)	
Screening	52 (3.58)	1 (3.70)	20 (4.47)	13 (2.29)	18 (4.38)	
TNM stage at diagnosis^†^						
I	239 (16.45)	3 (21.43)	70 (22.08)	95 (21.69)	71 (22.12)	0.681
II	246 (16.93)	4 (28.57)	79 (24.92)	101 (23.06)	62 (19.31)	
III	361 (24.84)	2 (14.29)	98 (30.91)	149 (34.02)	112 (34.89)	
IV	244 (16.79)	5 (35.71)	70 (22.08)	93 (21.23)	76 (23.68)	
Missing‡	363 (24.98)	13 (48.15)	130 (29.08)	130 (22.89)	90 (21.90)	

BMI: underweight <18.5 kg/m2, normal weight 18.5–24.9 kg/m2, overweight 25–29.9 kg/m2, obese 30 kg/m2.

* χ2 p value

† Percentages for stages I to IV are for the subgroup of patients with non-missing TNM stage at diagnosis

‡ Precentages for missing TNM stage at diagnosis refer to the total sample

BMI, body mass index; CIBH, change in bowel habit; CRC, colorectal cancer; GI, gastrointestinal; GP, general practitioner; RB, rectal bleeding; TNM, tumour, node, metastases.

### Emergency cancer diagnosis by BMI category

**Figure 2:**
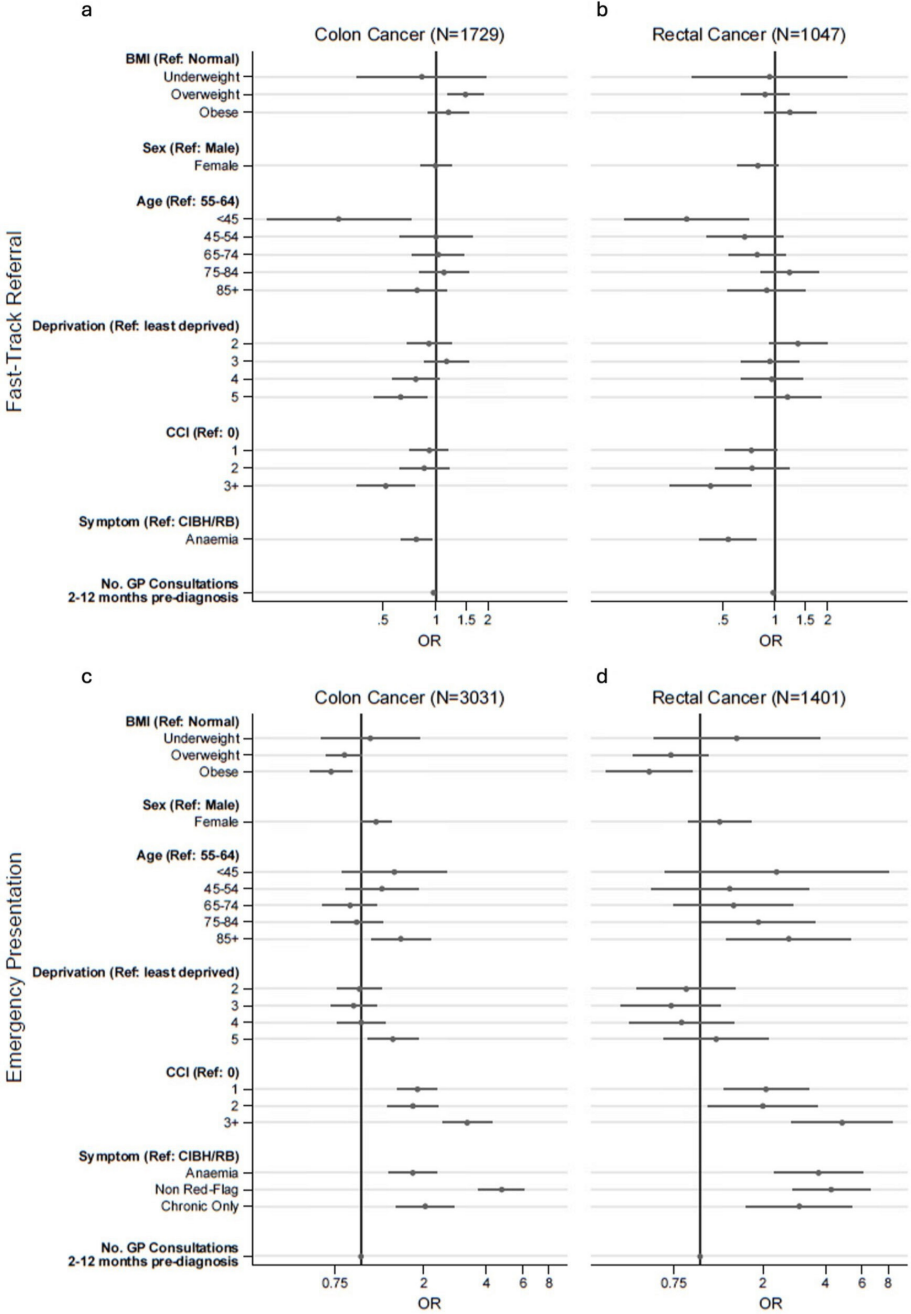
Multivariable logistic regression ORs for the association between BMI, sociodemographic and clinical characteristics and fast-track referrals (**a and b**) emergency presentation (**c and d**). Plots showing the adjusted ORs and 95% CIs for the association between BMI, main outcome variables and sociodemographic and clinical characteristics. For the two main outcome variables, fast-track referral (**a, b**) and emergency presentation (**c, d**), results are shown separately for colon and rectal cancer. All models were adjusted for relevant covariates as described in the Methods section. BMI, body mass index; CCI, Charlson Comorbidity Index; CIBH, change in bowel habit; RB, rectal bleeding.

Both patients with colon and rectal cancer with obesity, compared with those with normal weight, had lower risk of being diagnosed following EP (colon 23.6% vs 32.1%; fully adjusted OR: 0.72, p=0.005, 95% CI 0.57 to 0.90; rectum 8.3% vs 14.8%; OR: 0.57, p=0.021, 95% CI 0.35 to 0.92) (table 2, figure 2a,b). Similarly, patients with colon cancer in the overweight group, compared with normal weight, had lower odds of EP in the partially adjusted analysis (controlling for sex, age and deprivation) (26.7% vs 32.1%; partially adjusted OR: 0.81, p=0.032, 95% CI 0.67 to 0.98), while the association was not significant in the fully adjusted model (OR: 0.83, p=0.068, 95% CI 0.68 to 1.01). For rectal cancer, overweight was not significantly associated with EP in adjusted models (figure 2, online supplemental table 2).

There was no evidence of interactions between BMI and sex, age or deprivation (likelihood ratio tests all p>0.07).

### Fast-track referral by BMI category

Analysis of the patient subgroup with new-onset change in bowel habit/rectal bleeding or anaemia indicated that overweight (but not obesity), compared with normal weight, was associated with a higher likelihood of fast-track referral for colon cancer (fully adjusted OR:1.48, p=0.001, 95% CI 1.16 to 1.88) (figure 2c). There was no evidence for an association between BMI category and fast-track referrals for patients with rectal cancer (figure 2d) (online supplemental table 3).

### Sensitivity analysis

There was little variation in results when comparing fully adjusted models using individual comorbidities instead of CCI (online supplemental table 4) or using BMI measurements taken within 6 or 3 years prior to diagnosis (online supplemental table 5). Individuals with missing BMI were younger, less deprived, and had fewer comorbidities (online supplemental table 6). Patients with colon cancer with missing BMI also had a lower percentage of endoscopy and a higher percentage of EP (online supplemental table 7). In fully adjusted regression analysis, missing BMI was associated with increased odds of EP compared with normal weight for colon (OR: 1.50, p=0.001, 95% CI 1.18 to 1.89) but not rectal cancer (OR: 1.17, p=0.494, 95% CI 0.74 to 1.84) (online supplemental table 5).

## Discussion

Our study provides the first population-based retrospective cohort study to investigate the association between BMI, red-flag symptoms, endoscopy use and emergency CRC diagnosis. Patients with rectal cancer with obesity and overweight versus those of normal weight were more likely to present with red-flag symptoms, such as a change in bowel habit and rectal bleeding. Endoscopy was used more frequently in patients with obesity compared with those of normal weight in the year pre-cancer diagnosis. Among patients presenting with red flag symptoms, those with overweight had 50% higher odds of being referred urgently compared with those of normal weight. The odds of emergency CRC diagnosis were substantially lower for patients with obesity compared with normal weight.

### Interpretation and comparison with the literature

Our study suggests that symptom presentation before CRC diagnosis varies by BMI group, which may partially contribute to BMI-related differences in diagnostic route. In our study, patients with rectal cancer with obesity had more frequent primary care records of red-flag symptoms, which are associated with reduced EP risk.^12 36^

More frequent red flag symptoms among this group may arise due to the association between obesity and gastrointestinal diseases, for example, haemorrhoids or diverticular diseases, which can also present with similar symptoms, including rectal bleeding or change in bowel habit.^37^ Increased GP presentation with red flag symptoms, whether driven by CRC or gastrointestinal diseases, may provide additional opportunities for non-emergency diagnosis of an as-yet undetected cancer among higher BMI groups. This may also explain the observed higher endoscopy use, also associated with reduced EP risk,^38^,^40^ in the year pre-diagnosis among patients with obesity/overweight compared with normal weight. Higher rates of red flag symptoms among patients with obesity may also influence patient help-seeking behaviour, and could also contribute to differences in other routes to cancer diagnosis not examined here, such as increased engagement with CRC screening programmes.^41^

However, reduced EP risk among patients with obesity remained when controlling for increased reporting of red flag symptoms among this group. This could reflect limitations of the symptom data available, which lacks complexity regarding severity or duration, or differences in symptom reporting, both of which could vary by BMI. It may also point to non-symptom related explanations for lower EP risk among those with obesity, such as variations in clinical decision-making related to BMI. GP awareness of high BMI as a CRC risk factor may lead to increased referrals for endoscopies for patients with overweight or obesity, as observed in our results.

Although overweight was associated with a higher likelihood of fast-track referrals, we did not find clear evidence for obesity. These findings suggest that while GPs may want to exclude cancer in patients with obesity, they may not consider it to be urgent and referrals for endoscopies are made through non-fast-track pathways. However, referral for endoscopy through non-fast-track pathways does not seem to be associated with a higher risk of emergency diagnosis among patients with obesity. On the contrary, our findings show a lower risk of emergency diagnosis in patients with obesity compared with normal weight, in line with work from Canada and Scotland reporting a lower proportion of EPs in patients with obesity (BMI 30–34.9 kg/m^2^)^22^ or visceral obesity.^23^ The highest risk of emergency diagnosis was previously reported among those with morbid obesity (BMI>35 kg/m^2^), but small sample size in our study meant this group could not be analysed separately.

### Strengths and limitations

The use of high-quality, nationally representative datasets^29 42^ is a strength of the study. Particularly, the use of NCRAS, which includes all individuals diagnosed with cancer in England.^30^ Data in CPRD and HES are collected prospectively, limiting the risk of recall bias.

A limitation of the study is the missing BMI data. Weight measurements are not always taken in primary care, with some patients, including those with high or low BMI, being more likely to have a recent measurement, while normal weight patients might be more likely to have missing weight measurements.^34^ Sensitivity analyses showed that, when compared with other BMI categories, patients with missing BMI had increased odds of EP for colon cancer. This suggests a possible underestimation of EP for normal weight patients with colon cancer, considering that some of these patients are more likely to be included in the missing BMI category. The missing not at random nature of BMI also meant that standard multiple imputation of BMI could not be used.^43^ We did not examine the stage at diagnosis, and future studies could investigate if patients with obesity also have a lower risk of advanced stage cancer.

### Implications for research and/or practice

The findings need replication in other study settings and data sources. Qualitative research will also be useful to identify the role of BMI on GP suspicion of cancer and clinical decision-making regarding referrals for diagnostic investigations. Although high BMI was not related to less timely cancer diagnosis here, previous work suggests this could occur in some cases through the ‘alternative explanations’ mechanism, as people with overweight/obesity are more likely to attribute CRC red flag symptoms to another cause.^44^ In recognition of varying potential impacts of BMI, future work should focus on investigating the relationship between BMI, patient help-seeking behaviour, clinical decision making and route to diagnosis.

Given the increasing pressure on UK endoscopy services and the additional impact of COVID-19^45^ on waiting times for endoscopy, it would be important to have access to up-to-date linked primary care, secondary care and cancer registration data. This would allow an examination of whether patients at higher risk of cancer, such as those with obesity, can access diagnostic services in a timely fashion to diagnose cancer promptly and prevent emergency cancer diagnoses.

## Conclusion

Our findings suggest BMI may play an important role in route to rectal cancer diagnosis, potentially influencing both symptomatic presentation and clinical decision making regarding the use of relevant diagnostic tests. Future research to understand how BMI impacts route to cancer diagnosis is needed to identify patient groups and points during the diagnostic and care pathways that may benefit from targeted intervention to improve cancer diagnosis and outcomes.

## Supplementary material

10.1136/bmjopen-2025-107468 online supplemental file 1

## Data Availability

No data are available.

## References

[1] Hamilton W, Walter FM, Rubin G (2016). Improving early diagnosis of symptomatic cancer. Nat Rev Clin Oncol.

[2] Department of Health and Social Care (2018). Government announces plans for earlier diagnosis for cancer patients. https://www.gov.uk/government/news/government-announces-plans-for-earlier-diagnosis-for-cancer-patients.

[3] Zhou Y, Abel GA, Hamilton W (2017). Diagnosis of cancer as an emergency: a critical review of current evidence. Nat Rev Clin Oncol.

[4] McPhail S, Elliss-Brookes L, Shelton J (2013). Emergency presentation of cancer and short-term mortality. Br J Cancer.

[5] Barclay M (2016). Factors Affecting Short-term Mortality in Women With Ovarian, Tubal, or Primary Peritoneal Cancer: Population-Based Cohort Analysis of English National Cancer Registration Data. Int J Gynecol Cancer Off J Int Gynecol Cancer Soc.

[6] Comber H, Sharp L, de Camargo Cancela M (2016). Causes and outcomes of emergency presentation of rectal cancer. Int J Cancer.

[7] Danckert B, Falborg AZ, Christensen NL (2021). Routes to diagnosis and the association with the prognosis in patients with cancer - A nationwide register-based cohort study in Denmark. Cancer Epidemiol.

[8] Salika T, Abel GA, Mendonca SC (2018). Associations between diagnostic pathways and care experience in colorectal cancer: evidence from patient-reported data. Frontline Gastroenterol.

[9] Pham TM, Gomez-Cano M, Salika T (2019). Diagnostic route is associated with care satisfaction independently of tumour stage: Evidence from linked English Cancer Patient Experience Survey and cancer registration data. Cancer Epidemiol.

[10] Goodyear SJ, Leung E, Menon A (2008). The effects of population-based faecal occult blood test screening upon emergency colorectal cancer admissions in Coventry and north Warwickshire. Gut.

[11] Abel GA, Shelton J, Johnson S (2015). Cancer-specific variation in emergency presentation by sex, age and deprivation across 27 common and rarer cancers. Br J Cancer.

[12] Renzi C, Lyratzopoulos G, Card T (2016). Do colorectal cancer patients diagnosed as an emergency differ from non-emergency patients in their consultation patterns and symptoms? A longitudinal data-linkage study in England. Br J Cancer.

[13] Pennisi F, Buzzoni C, Russo AG (2025). Comorbidities, Socioeconomic Status, and Colorectal Cancer Diagnostic Route. *JAMA Netw Open*.

[14] NHS Digital (2020). Statistics on obesity, physical activity and diet,England. https://digital.nhs.uk/data-and-information/publications/statistical/statistics-on-obesity-physical-activity-and-diet/england-2020.

[15] Dai Z, Xu YC, Niu L (2007). Obesity and colorectal cancer risk: a meta-analysis of cohort studies. World J Gastroenterol.

[16] Moghaddam AA, Woodward M, Huxley R (2007). Obesity and risk of colorectal cancer: a meta-analysis of 31 studies with 70,000 events. Cancer Epidemiol Biomarkers Prev.

[17] Ma Y, Yang Y, Wang F (2013). Obesity and Risk of Colorectal Cancer: A Systematic Review of Prospective Studies. PLoS ONE.

[18] Ning Y, Wang L, Giovannucci EL (2010). A quantitative analysis of body mass index and colorectal cancer: findings from 56 observational studies. Obes Rev.

[19] Parkin E, O’Reilly DA, Sherlock DJ (2014). Excess adiposity and survival in patients with colorectal cancer: a systematic review. Obes Rev.

[20] Wu S, Liu J, Wang X (2014). Association of obesity and overweight with overall survival in colorectal cancer patients: a meta-analysis of 29 studies. Cancer Causes Control.

[21] Lee J, Meyerhardt JA, Giovannucci E (2015). Association between Body Mass Index and Prognosis of Colorectal Cancer: A Meta-Analysis of Prospective Cohort Studies. PLoS ONE.

[22] Mitchell AD, Inglis KM, Murdoch JM (2007). Emergency room presentation of colorectal cancer: a consecutive cohort study. Ann Surg Oncol.

[23] Golder AM, Sin LKE, Alani F (2022). The relationship between the mode of presentation, CT-derived body composition, systemic inflammatory grade and survival in colon cancer. J Cachexia Sarcopenia Muscle.

[24] Xiao H, Tan F, Goovaerts P (2016). Impact of Comorbidities on Prostate Cancer Stage at Diagnosis in Florida. Am J Mens Health.

[25] Fleming ST, Pursley HG, Newman B (2005). Comorbidity as a predictor of stage of illness for patients with breast cancer. Med Care.

[26] Gurney J, Sarfati D, Stanley J (2015). The impact of patient comorbidity on cancer stage at diagnosis. *Br J Cancer*.

[27] Renzi C, Kaushal A, Emery J (2019). Comorbid chronic diseases and cancer diagnosis: disease-specific effects and underlying mechanisms. Nat Rev Clin Oncol.

[28] Elliss-Brookes L, McPhail S, Ives A (2012). Routes to diagnosis for cancer - determining the patient journey using multiple routine data sets. Br J Cancer.

[29] Henson KE, Elliss-Brookes L, Coupland VH (2020). Data Resource Profile: National Cancer Registration Dataset in England. Int J Epidemiol.

[30] Padmanabhan S, CPRD (2015). CPRD gold data specification.

[31] Majano SB, Lyratzopoulos G, Rachet B (2022). Do presenting symptoms, use of pre-diagnostic endoscopy and risk of emergency cancer diagnosis vary by comorbidity burden and type in patients with colorectal cancer?. Br J Cancer.

[32] Benitez Majano S, Lyratzopoulos G, de Wit NJ (2022). Mental Health Morbidities and Time to Cancer Diagnosis Among Adults With Colon Cancer in England. *JAMA Netw Open*.

[33] NICE (2021). Symptoms suggestive of gastrointestinal tract (lower) cancers. https://cks.nice.org.uk/topics/gastrointestinal-tract-lower-cancers-recognition-referral/diagnosis/symptoms-suggestive-of-gastrointestinal-tract-lower-cancers/.

[34] Nicholson BD, Aveyard P, Bankhead CR (2019). Determinants and extent of weight recording in UK primary care: an analysis of 5 million adults’ electronic health records from 2000 to 2017. BMC Med.

[35] Sundararajan V, Henderson T, Perry C (2004). New ICD-10 version of the Charlson comorbidity index predicted in-hospital mortality. J Clin Epidemiol.

[36] Singh H, Daci K, Petersen LA (2009). Missed opportunities to initiate endoscopic evaluation for colorectal cancer diagnosis. Am J Gastroenterol.

[37] Emerenziani S, Pier Luca Guarino M, Trillo Asensio L (2020). Role of Overweight and Obesity in Gastrointestinal Disease. Nutrients.

[38] Rabeneck L, Paszat LF, Li C (2006). Risk factors for obstruction, perforation, or emergency admission at presentation in patients with colorectal cancer: a population-based study. Am J Gastroenterol.

[39] Pruitt SL, Davidson NO, Gupta S (2014). Missed opportunities: racial and neighborhood socioeconomic disparities in emergency colorectal cancer diagnosis and surgery. BMC Cancer.

[40] Gunnarsson H, Jennische K, Forssell S (2014). Heterogeneity of colon cancer patients reported as emergencies. World J Surg.

[41] Golder AM, Mshihadani A, McMillan DC (2022). Route to diagnosis of colorectal cancer and association with survival within the context of a bowel screening programme. Public Health (Fairfax).

[42] Herrett E, Gallagher AM, Bhaskaran K (2015). Data Resource Profile: Clinical Practice Research Datalink (CPRD). Int J Epidemiol.

[43] White IR, Royston P, Wood AM (2011). Multiple imputation using chained equations: Issues and guidance for practice. Stat Med.

[44] Ricciardi GE, Pennisi F, Von Wagner C (2025). Attribution of colorectal cancer symptoms to medications for pre-existing chronic conditions: a secondary analysis of a vignette study in England. J Public Health (Oxf).

[45] Ravindran S, Bassett P, Shaw T (2021). National census of UK endoscopy services in 2019. Frontline Gastroenterol.

[46] Drury CAA, Louis M (2002). Exploring the association between body weight, stigma of obesity, and health care avoidance. J Am Acad Nurse Pract.

[47] Lee JA, Pausé CJ (2063). Stigma in Practice: Barriers to Health for Fat Women. Front Psychol.

[48] Rubino F, Puhl RM, Cummings DE (2020). Joint international consensus statement for ending stigma of obesity. Nat Med.

[49] National Cancer Institute (2025). Obesity and cancer fact sheet. https://www.cancer.gov/about-cancer/causes-prevention/risk/obesity/obesity-fact-sheet.

[50] Alemán JO, Eusebi LH, Ricciardiello L (2014). Mechanisms of obesity-induced gastrointestinal neoplasia. Gastroenterology.

[51] Brändstedt J, Wangefjord S, Borgquist S (2013). Influence of anthropometric factors on tumour biological characteristics of colorectal cancer in men and women: a cohort study. J Transl Med.

