# Supplementary material for: Variations in symptoms, endoscopy use and emergency diagnosis of colorectal cancer by body mass index: a retrospective cohort study using linked electronic health records in England
Source: BMJ Open. 2026 May 7;16(5):e107468. doi: 10.1136/bmjopen-2025-107468 (PMC13157786; doi:10.1136/bmjopen-2025-107468)
Supplement: online supplemental file 1 [file bmjopen-16-5-s001.docx]

Supplementary Material

| Box S1: CRC-relevant sign and symptoms | |
| --- | --- |
| Rectal Bleeding (CIBH) | Red-Flag Symptoms |
| Change in Bowel Habits (RB) |  |
| Anaemia (Laboratory Confirmed) | Red-Flag Sign |
| Abdominal Pain | Non Red-Flag Symptoms |
| Constipation |  |
| Diarrhoea |  |
| Weight Loss |  |
| Fatigue |  |
| Jaundice |  |
| Tenesmus |  |
| Bloating |  |
| Lump Mass |  |
| Obstruction |  |

| Box S2: 17 Comorbidities used to calculate Charlson Comorbidity Index (CCI) score |
| --- |
| Myocardial infarction  Congestive heart failure  Peripheral vascular disease  Cerebrovascular disease  Dementia  COPD  Rheumatic disease  Peptic ulcer disease  Mild liver disease  Diabetes without chronic complications  Diabetes with chronic complications  Hemiplegia or paraplegia  Renal disease  Moderate or severe liver disease  AIDS/HIV  Any malignancy  Metastatic solid tumours |
| Comorbidities were classed as present if they were recorded in HES within 6 years pre-CRC diagnosis. CCI score was analysed by categories (0, 1, 2, 3+). |

| Table S1: Number of primary care appointments 2-12 months pre-diagnosis | | | | | | | |
| --- | --- | --- | --- | --- | --- | --- | --- |
|  | All Patients | Underweight | Normal Weight | Overweight | Obese |  | P-value^2^ |
|  |  |  |  |  |  |  |  |
| **Colon Cancer** | N=3137 |  |  |  |  |  |  |
| Mean | 18.31 | 23.90 | 19.72 | 18.53 | 19.04 |  |  |
| Median | 15 | 22 | 16 | 16 | 16 |  |  |
| Interquartile Range | 9,24 | 13,32 | 9,26 | 9,25 | 9,26 |  |  |
| P-Value^1^ |  | **0.0016** |  | 0.377 | 0.589 |  |  |
|  |  |  |  |  |  |  |  |
| Categorical Number of Visits | |  |  |  |  |  |  |
| 0 | 77 (1.68) | 1 (1.39) | 22 (2.19) | 23 (1.89) | 31 (3.67) |  | 0.144 |
| 1-4 | 225 (4.90) | 2 (2.78) | 71 (7.08) | 95 (7.80) | 57 (6.75) |  |  |
| 5-9 | 500 (10.89) | 7 (9.72) | 164 (16.35) | 193 (15.85) | 136 (16.11) |  |  |
| 10+ | 2335 (50.89) | 62 (86.11) | 746 (74.38) | 907 (74.47) | 620 (73.46) |  |  |
|  |  |  |  |  |  |  |  |
|  |  |  |  |  |  |  |  |
| **Rectal Cancer** | N=1453 |  |  |  |  |  |  |
| Mean | 16.20 | 21.67 | 16.58 | 15.50 | 16.38 |  |  |
| Median | 12 | 21 | 13 | 13 | 14 |  |  |
| Interquartile Range | 6,20 | 13,32 | 8,22 | 7,20 | 7,22 |  |  |
| P-Value^1^ |  | **0.008** |  | 0.423 | 0.926 |  |  |
|  |  |  |  |  |  |  |  |
| Categorical Number of Visits | |  |  |  |  |  |  |
| 0 | 23 (0.50) | 0 (0.00) | 8 (1.79) | 8 (1.41) | 7 (1.70) |  | 0.378 |
| 1-4 | 178 (3.88) | 2 (7.41) | 52 (11.63) | 77 (13.56) | 47 (11.44) |  |  |
| 5-9 | 303 (6.60) | 1 (3.70) | 102 (22.82) | 114 (20.07) | 86 (20.92) |  |  |
| 10+ | 949 (20.70) | 24 (88.89) | 285 (63.76) | 369 (64.96) | 271 (65.94) |  |  |
| ^1^P-value for Wilcoxon rank sum test comparing median appointment number for each group to the normal weight group  ^2^Chi^2^ test  *BMI* Body Mass index; underweight <18.5 kg/m^2^, normal weight 18.5-24.9 kg/m^2^, overweight 25-29.9 kg/m^2^, obese $\geq$30 kg/m^2^ | | | | | | | |

| Table S2: Multivariate Logistic Regression: Odds Ratios of Emergency Presentation for Unadjusted, Partially Adjusted and Fully Adjusted Models | | | | | | | | | |  |
| --- | --- | --- | --- | --- | --- | --- | --- | --- | --- | --- |
|  | Model 1 | | | Model 2 | | | Model 3 | | |  |
|  | OR | 95%CI | p | OR | 95%CI | P | OR | 95%CI | p |  |
| **COLON CANCER** N=3031 |  |  |  |  |  |  |  |  |  |  |
| **BMI** |  |  |  |  |  |  |  |  |  |  |
| Underweight | 1.41 | 0.86,2.30 | 0.172 | 1.34 | 0.81,2.23 | 0.253 | 1.11 | 0.64,1.91 | 0.714 |  |
| Normal Weight | REF |  |  | REF |  |  | REF |  |  |  |
| Overweight | 0.78 | 0.65,0.93 | **0.007** | 0.81 | 0.67,0.98 | **0.032** | 0.83 | 0.68,1.01 | 0.068 |  |
| Obese | 0.67 | 0.55,0.83 | **<0.001** | 0.73 | 0.59,0.91 | **0.004** | 0.72 | 0.57,0.90 | **0.005** |  |
|  |  |  |  |  |  |  |  |  |  |  |
| **Sex** |  |  |  |  |  |  |  |  |  |  |
| Female |  |  |  | 1.11 | 0.94,1.30 | 0.224 | 1.18 | 1.00,1.40 | 0.057 |  |
| Male |  |  |  | REF |  |  | REF |  |  |  |
|  |  |  |  |  |  |  |  |  |  |  |
| **Age** |  |  |  |  |  |  |  |  |  |  |
| <45 |  |  |  | 1.43 | 0.81,2.50 | 0.214 | 1.44 | 0.82,2.56 | 0.207 |  |
| 45-54 |  |  |  | 1.20 | 0.81,1.76 | 0.366 | 1.26 | 0.84,1.88 | 0.265 |  |
| 55-64 |  |  |  | REF |  |  | REF |  |  |  |
| 65-74 |  |  |  | 0.96 | 0.73,1.28 | 0.799 | 0.89 | 0.66,1.19 | 0.424 |  |
| 75-84 |  |  |  | 1.09 | 0.84,1.42 | 0.528 | 0.95 | 0.71,1.27 | 0.737 |  |
| 85+ |  |  |  | 1.88 | 1.40,2.51 | **<0.001** | 1.55 | 1.12,2.14 | **0.008** |  |
|  |  |  |  |  |  |  |  |  |  |  |
| **Deprivation Quintile** |  |  |  |  |  |  |  |  |  |  |
| 1 (Least Deprived) |  |  |  | REF |  |  | REF |  |  |  |
| 2 |  |  |  | 1.03 | 0.82,1.31 | 0.787 | 0.98 | 0.77,1.27 | 0.886 |  |
| 3 |  |  |  | 0.92 | 0.72,1.17 | 0.499 | 0.92 | 0.72,1.18 | 0.516 |  |
| 4 |  |  |  | 1.10 | 0.86,1.40 | 0.468 | 1.00 | 0.77,1.30 | 0.997 |  |
| 5 (Most Deprived) |  |  |  | 1.64 | 1.27,2.13 | **<0.001** | 1.42 | 1.08,1.87 | **0.012** |  |
|  |  |  |  |  |  |  |  |  |  |  |
| **CCI** |  |  |  |  |  |  |  |  |  |  |
| 0 |  |  |  |  |  |  | REF |  |  |  |
| 1 |  |  |  |  |  |  | 1.87 | 1.50,2.31 | **<0.001** |  |
| 2 |  |  |  |  |  |  | 1.77 | 1.35,2.34 | **<0.001** |  |
| 3+ |  |  |  |  |  |  | 3.23 | 2.47,4.23 | **<0.001** |  |
|  |  |  |  |  |  |  |  |  |  |  |
| **New Onset Symptom** |  |  |  |  |  |  |  |  |  |  |
| CIBH/RB |  |  |  |  |  |  | REF |  |  |  |
| Anaemia |  |  |  |  |  |  | 1.77 | 1.37,2.29 | **<0.001** |  |
| Non Red-Flag |  |  |  |  |  |  | 4.74 | 3.69,6.09 | **<0.001** |  |
| Only Chronic |  |  |  |  |  |  | 2.03 | 1.48,2.80 | **<0.001** |  |
|  |  |  |  |  |  |  |  |  |  |  |
| **No. Consultations** |  |  |  |  |  |  | 0.998 | 0.99,1.01 | 0.631 |  |
| **RECTAL CANCER** (N=1401) |  |  |  |  |  |  |  |  |  |  |
| **BMI** |  |  |  |  |  |  |  |  |  |  |
| Underweight | 2.43 | 1.01,5.82 | **0.046** | 1.87 | 0.73,4.74 | 0.190 | 1.50 | 0.60,3.74 | 0.387 |  |
| Normal Weight | REF |  |  | REF |  |  | REF |  |  |  |
| Overweight | 0.65 | 0.45,0.95 | **0.025** | 0.73 | 0.50,1.07 | 0.111 | 0.72 | 00.48,1.10 | 0.127 |  |
| Obese | 0.52 | 0.33,0.80 | **0.003** | 0.61 | 0.39,0.97 | **0.038** | 0.57 | 0.35,0.92 | **0.021** |  |
|  |  |  |  |  |  |  |  |  |  |  |
| **Sex** |  |  |  |  |  |  |  |  |  |  |
| Female |  |  |  | 1.21 | 0.87,1.70 | 0.264 | 1.24 | 0.87,1.76 | 0.228 |  |
| Male |  |  |  | REF |  |  | REF |  |  |  |
|  |  |  |  |  |  |  |  |  |  |  |
| **Age** |  |  |  |  |  |  |  |  |  |  |
| <45 |  |  |  | 1.81 | 0.54,6.01 | 0.335 | 2.33 | 0.68,8.00 | 0.180 |  |
| 45-54 |  |  |  | 1.18 | 0.49,2.76 | 0.725 | 1.39 | 0.58.3.31 | 0.461 |  |
| 55-64 |  |  |  | REF |  |  | REF |  |  |  |
| 65-74 |  |  |  | 1.61 | 0.85,3.03 | 0.141 | 1.45 | 0.75,2.79 | 0.270 |  |
| 75-84 |  |  |  | 2.58 | 1.43,4.67 | **0.002** | 1.90 | 1.02,3.56 | **0.044** |  |
| 85+ |  |  |  | 4.79 | 2.54,9.03 | **<0.001** | 2.67 | 1.34,5.31 | **0.005** |  |
|  |  |  |  |  |  |  |  |  |  |  |
| **Deprivation Quintile** |  |  |  |  |  |  |  |  |  |  |
| 1 (Least Deprived) |  |  |  | REF |  |  | REF |  |  |  |
| 2 |  |  |  | 1.02 | 0.61,1.71 | 0.941 | 0.86 | 0.50,1.48 | 0.579 |  |
| 3 |  |  |  | 0..92 | 0.54,1.54 | 0.738 | 0.73 | 0.42,1.26 | 0.253 |  |
| 4 |  |  |  | 1.02 | 0.59,1.77 | 0.932 | 0.81 | 0.461.45 | 0.483 |  |
| 5 (Most Deprived) |  |  |  | 1.67 | 0.99,2.79 | **0.053** | 1.19 | 0.67,2.12 | 0.546 |  |
|  |  |  |  |  |  |  |  |  |  |  |
| **CCI** |  |  |  |  |  |  |  |  |  |  |
| 0 |  |  |  |  |  |  | REF |  |  |  |
| 1 |  |  |  |  |  |  | 2.07 | 1.29,3.31 | **0.002** |  |
| 2 |  |  |  |  |  |  | 2.00 | 1.09,3.67 | **0.025** |  |
| 3+ |  |  |  |  |  |  | 4.81 | 2.76,8.37 | **<0.001** |  |
|  |  |  |  |  |  |  |  |  |  |  |
| **New Onset Symptom** |  |  |  |  |  |  |  |  |  |  |
| CIBH/RB |  |  |  |  |  |  | REF |  |  |  |
| Anaemia |  |  |  |  |  |  | 3.70 | 2.27,6.05 | **<0.001** |  |
| Non Red-Flag |  |  |  |  |  |  | 4.26 | 2.77,6.54 | **<0.001** |  |
| Chronic Only |  |  |  |  |  |  | 2.99 | 1.67,5.37 | **<0.001** |  |
|  |  |  |  |  |  |  |  |  |  |  |
| **No. Consultations** |  |  |  |  |  |  | 0.999 | 0.99,1.01 | 0.837 |  |
| Model 1 – Unadjusted: Odds Ratios for Emergency Presentation by BMI category  Model 2 – Partially adjusted: Controlling for socio-demographic characteristics  Model 3 – Fully adjusted: Controlling for sociodemographic characteristics, comorbidities, symptoms and number of primary care consultations 2-12 months pre-diagnosis  *BMI* Body Mass index; underweight <18.5 kg/m^2^, normal weight 18.5-24.9 kg/m^2^, overweight 25-29.9 kg/m^2^, obese $\geq$30 kg/m^2^*; CCI* Charlson Comorbidity Index; *CIBH* change in bowel habit; *RB* rectal bleeding | | | | | | | | | |  |

| Table S3: Multivariate Logistic Regression: Odds Ratios of Fast-Track referral among patients with new onset red-flag symptoms/signs (CIBH/RB or anaemia) for Unadjusted, Partially-Adjusted and Fully-Adjusted Models | | | | | | | | | |
| --- | --- | --- | --- | --- | --- | --- | --- | --- | --- |
|  | Model 1 | | | Model 2 | | | Model 3 | | |
|  | OR | 95%CI | p | OR | 95%CI | P | OR | 95%CI | p |
| **COLON CANCER** (N=1729) |  |  |  |  |  |  |  |  |  |
| **BMI** |  |  |  |  |  |  |  |  |  |
| Underweight | 0.65 | 0.30,1.43 | 0.282 | 0.71 | 0.32,1.55 | 0.384 | 0.83 | 0.36,1.95 | 0.672 |
| Normal Weight | REF |  |  | REF |  |  | REF |  |  |
| Overweight | 1.49 | 1.18,1.87 | **0.001** | 1.44 | 1.14,1.83 | **0.002** | 1.48 | 1.16,1.88 | **0.001** |
| Obese | 1.20 | 0.93,1.54 | 0.166 | 1.12 | 0.87,1.46 | 0.383 | 1.18 | 0.90,1.55 | 0.221 |
|  |  |  |  |  |  |  |  |  |  |
| **Sex** |  |  |  |  |  |  |  |  |  |
| Female |  |  |  | 0.96 | 0.79,1.17 | 0.684 | 1.00 | 0.81,1.22 | 0.976 |
| Male |  |  |  | REF |  |  | REF |  |  |
|  |  |  |  |  |  |  |  |  |  |
| **Age** |  |  |  |  |  |  |  |  |  |
| <45 |  |  |  | 0.30 | 0.12,0.77 | **0.013** | 0.28 | 0.11,0.72 | **0.008** |
| 45-54 |  |  |  | 1.04 | 0.65,1.65 | 0.881 | 1.00 | 0.62,1.62 | 0.989 |
| 55-64 |  |  |  | REF |  |  | REF |  |  |
| 65-74 |  |  |  | 0.88 | 0.63,1.22 | 0.444 | 1.03 | 0.74,1.45 | 0.989 |
| 75-84 |  |  |  | 0.80 | 0.59,1.09 | 0.166 | 1.11 | 0.81,1.53 | 0.504 |
| 85+ |  |  |  | 0.51 | 0.35,0.74 | **<0.001** | 0.78 | 0.53,1.16 | 0.219 |
|  |  |  |  |  |  |  |  |  |  |
| **Deprivation Quintile** |  |  |  |  |  |  |  |  |  |
| 1 (Least Deprived) |  |  |  | REF |  |  | REF |  |  |
| 2 |  |  |  | 0.91 | 0.68,1.21 | 0.498 | 0.91 | 0.68,1.23 | 0.551 |
| 3 |  |  |  | 1.17 | 0.88,1.54 | 0.282 | 1.15 | 0.87,1.54 | 0.327 |
| 4 |  |  |  | 0.76 | 0.56,1.04 | 0.085 | 0.77 | 0.56,1.05 | 0.098 |
| 5 (Most Deprived) |  |  |  | 0.58 | 0.42,0.82 | **0.002** | 0.63 | 0.44,0.90 | **0.010** |
|  |  |  |  |  |  |  |  |  |  |
| **CCI** |  |  |  |  |  |  |  |  |  |
| 0 |  |  |  |  |  |  | REF |  |  |
| 1 |  |  |  |  |  |  | 0.92 | 0.71,1.18 | 0.506 |
| 2 |  |  |  |  |  |  | 0.86 | 0.62,1.19 | 0.355 |
| 3+ |  |  |  |  |  |  | 0.52 | 0.36,0.75 | **<0.001** |
|  |  |  |  |  |  |  |  |  |  |
| **New Onset Symptom** |  |  |  |  |  |  |  |  |  |
| CIBH/RB |  |  |  |  |  |  | REF |  |  |
| Anaemia |  |  |  |  |  |  | 0.77 | 0.63,0.95 | **0.013** |
|  |  |  |  |  |  |  |  |  |  |
| **No. GP Consultations^2^** |  |  |  |  |  |  | 0.97 | 0.96,0.98 | **<0.001** |
|  |  |  |  |  |  |  |  |  |  |
| **RECTAL CANCER**  (N=1047) |  |  |  |  |  |  |  |  |  |
| **BMI** |  |  |  |  |  |  |  |  |  |
| Underweight | 0.65 | 0.23,1.88 | 0.431 | 0.72 | 0.25,2.08 | 0.548 | 0.93 | 0.34,2.58 | 0.893 |
| Normal Weight | REF |  |  | REF |  |  | REF |  |  |
| Overweight | 0.95 | 0.71,1.28 | 0.749 | 0.91 | 0.67,1.22 | 0.519 | 0.88 | 0.64,1.20 | 0.413 |
| Obese | 1.22 | 0.89,1.67 | 0.223 | 1.16 | 0.83,1.60 | 0.384 | 1.22 | 0.87,1.71 | 0.249 |
|  |  |  |  |  |  |  |  |  |  |
| **Sex** |  |  |  |  |  |  |  |  |  |
| Female |  |  |  | 0.86 | 00.66,1.11 | 0.237 | 0.80 | 0.61,1.04 | 0.097 |
| Male |  |  |  | REF |  |  | REF |  |  |
|  |  |  |  |  |  |  |  |  |  |
| **Age** |  |  |  |  |  |  |  |  |  |
| <45 |  |  |  | 0.37 | 0.16,0.87 | **0.023** | 0.31 | 0.14,0.71 | **0.005** |
| 45-54 |  |  |  | 0.79 | 0.49,1.29 | 0.344 | 0.67 | 0.41,1.11 | 0.121 |
| 55-64 |  |  |  | REF |  |  | REF |  |  |
| 65-74 |  |  |  | 0.71 | 0.50,1.03 | 0.069 | 0.79 | 0.54,1.15 | 0.224 |
| 75-84 |  |  |  | 0.91 | 0.64,1.31 | 0.620 | 1.21 | 0.83,1.77 | 0.315 |
| 85+ |  |  |  | 0.58 | 0.36,0.95 | **0.031** | 0.90 | 0.54,1.50 | 0.681 |
|  |  |  |  |  |  |  |  |  |  |
| **Deprivation Quintile** |  |  |  |  |  |  |  |  |  |
| 1 (Least Deprived) |  |  |  | REF |  |  | REF |  |  |
| 2 |  |  |  | 1.25 | 0.86,1.81 | 0.238 | 1.36 | 0.93,1.98 | 0.118 |
| 3 |  |  |  | 0.89 | 0.62,1.29 | 0.546 | 0.94 | 0.64,1.36 | 0.733 |
| 4 |  |  |  | 0.88 | 0.60,1.30 | 0.515 | 0.96 | 0.64,1.43 | 0.841 |
| 5 (Most Deprived) |  |  |  | 0.93 | 0.62,1.41 | 0.740 | 1.18 | 0.76,1.83 | 0.453 |
|  |  |  |  |  |  |  |  |  |  |
| **CCI** |  |  |  |  |  |  |  |  |  |
| 0 |  |  |  |  |  |  | REF |  |  |
| 1 |  |  |  |  |  |  | 0.73 | 0.52,1.03 | 0.075 |
| 2 |  |  |  |  |  |  | 0.74 | 0.45,1.21 | 0.232 |
| 3+ |  |  |  |  |  |  | 0.43 | 0.25,0.73 | **0.002** |
|  |  |  |  |  |  |  |  |  |  |
| **New Onset Symptom** |  |  |  |  |  |  |  |  |  |
| CIBH/RB |  |  |  |  |  |  | REF |  |  |
| Anaemia |  |  |  |  |  |  | 0.54 | 0.37,0.79 | **0.001** |
|  |  |  |  |  |  |  |  |  |  |
| **No. GP Consultations^2^** |  |  |  |  |  |  | 0.98 | 0.96,0.99 | **<0.001** |
| Model 1 – Unadjusted: Odds Ratios for Two Week Wait Referral by BMI category  Model 2 – Partially adjusted: Controlling for socio-demographic characteristics  Model 3 – Fully adjusted: Controlling for sociodemographic characteristics, comorbidities, type of red-flag sign/symptoms and number of consultations  ^2^Number of primary care consultations in 2-12 months pre-diagnosis, OR for one additional consultation  *BMI* Body Mass index; underweight <18.5 kg/m^2^, normal weight 18.5-24.9 kg/m^2^, overweight 25-29.9 kg/m^2^, obese $\geq$30 kg/m^2^*; CCI* Charlson Comorbidity Index; *CIBH* change in bowel habit; *RB* rectal bleeding | | | | | | | | | |

| Table S4: Sensitivity Analysis - Odds Ratios of Emergency Presentation for Fully-Adjusted Multivariate Logistic Regression Models using Charlson Comorbidity Index Score or Individual Comorbidities | | | | | | | |
| --- | --- | --- | --- | --- | --- | --- | --- |
|  | | Colon Cancer | | | Rectal Cancer | | |
| Comorbidity | BMI Category | OR | 95%CI | P Value | OR | 95%CI | P Value |
|  |  |  |  |  |  |  |  |
| CCI | Underweight | 1.11 | 0.64,1.91 | 0.714 | 1.50 | 0.60,3.74 | 0.387 |
|  | Normal weight | REF |  |  | REF |  |  |
|  | Overweight | 0.83 | 0.68,1.01 | 0.068 | 0.72 | 0.48,1.10 | 0.127 |
|  | Obese | 0.72 | 0.57,0.90 | 0.005 | 0.57 | 0.35,0.92 | 0.021 |
|  |  |  |  |  |  |  |  |
| Diabetes | Underweight | 1.18 | 0.69,2.02 | 0.550 | 1.62 | 0.64,4.07 | 0.308 |
|  | Normal weight | REF |  |  | REF |  |  |
|  | Overweight | 0.83 | 0.68,1.00 | 0.054 | 0.76 | 0.51,1.14 | 0.183 |
|  | Obese | 0.73 | 0.58,0.92 | 0.007 | 0.60 | 0.37,0.96 | 0.032 |
|  |  |  |  |  |  |  |  |
| CVD | Underweight | 1.12 | 0.65,1.93 | 0.677 | 1.52 | 0.60,3.86 | 0.376 |
|  | Normal weight | REF |  |  | REF |  |  |
|  | Overweight | 0.81 | 0.67,0.99 | 0.037 | 0.78 | 0.52,1.17 | 0.222 |
|  | Obese | 0.76 | 0.60,0.95 | 0.017 | 0.62 | 0.38,0.99 | 0.044 |
|  |  |  |  |  |  |  |  |
| COPD | Underweight | 1.11 | 0.64,1.90 | 0.712 | 1.45 | 0.59,3.61 | 0.419 |
|  | Normal weight | REF |  |  | REF |  |  |
|  | Overweight | 0.83 | 0.69,1.01 | 0.069 | 0.76 | 0.51,1.14 | 0.184 |
|  | Obese | 0.76 | 0.61,0.95 | 0.016 | 0.64 | 0.40,1.01 | 0.057 |
| All models controlled for sex, age, deprivation, new onset symptom and number of primary care consultations 2-12 months pre-diagnosis  *BMI* Body Mass index; underweight <18.5 kg/m^2^, normal weight 18.5-24.9 kg/m^2^, overweight 25-29.9 kg/m^2^, obese $\geq$30 kg/m^2^*; CCI* Charlson Comorbidity Index; *CVD* cardiovascular disease (myocardial infarction, congestive heart failure, peripheral vascular disease, or cerebrovascular disease); *COPD* chronic obstructive pulmonary disease | | | | | | | |

| Table S5: Sensitivity Analysis – Odds ratios of Emergency Presentation from the final fully-adjusted Logistic Regression Model, a model using a 3-year BMI cut-off instead of the original 6-year cut-off and a model including patients with missing BMI | | | | | | | | | |
| --- | --- | --- | --- | --- | --- | --- | --- | --- | --- |
|  | Final Model | | | 3-year BMI cut-off | | | Final Model with Missing | | |
|  | OR | 95%CI | P | OR | 95%CI | P | OR | 95%CI | P |
| **COLON CANCER** | N=3031 |  |  | N=2515 |  |  | N=3610 |  |  |
| **BMI** |  |  |  |  |  |  |  |  |  |
| Underweight | 1.11 | 0.65,1.87 | 0.704 | 1.12 | 0.64,1.93 | 0.694 | 1.12 | 0.65,1.94 | 0.684 |
| Normal Weight | REF |  |  | REF |  |  | REF |  |  |
| Overweight | 0.83 | 0.68,1.01 | 0.069 | 0.89 | 0.71,1.11 | 0.288 | 0.83 | 0.68,1.01 | 0.067 |
| Obese | 0.72 | 0.57,0.90 | 0.004 | 0.73 | 0.57,0.94 | 0.031 | 0.73 | 0.58,0.91 | 0.006 |
| Missing |  |  |  |  |  |  | 1.50 | 1.18,1.89 | 0.001 |
| **RECTAL CANCER** | N=1401 |  |  | N=1181 |  |  | N=1733 |  |  |
| **BMI** |  |  |  |  |  |  |  |  |  |
| Underweight | 1.50 | 0.58,3.86 | 0.403 | 1.33 | 0.49,3.62 | 0.574 | 1.50 | 0.59,3.82 | 0.395 |
| Normal Weight | REF |  |  | REF |  |  | REF |  |  |
| Overweight | 0.72 | 0.48,1.09 | 0.123 | 0.63 | 0.41,0.98 | 0.043 | 0.73 | 0.48,1.11 | 0.139 |
| Obese | 0.57 | 0.35,0.92 | 0.020 | 0.45 | 0.27,0.76 | 0.003 | 0.57 | 0.35,0.92 | 0.021 |
| Missing |  |  |  |  |  |  | 1.17 | 0.74,1.84 | 0.494 |
| All models control for sex, age, deprivation, CCI category, new onset symptomatic presentation and number of primary care consultations in the 2-12 month pre-diagnosis  *BMI* Body Mass index; underweight <18.5 kg/m^2^, normal weight 18.5-24.9 kg/m^2^, overweight 25-29.9 kg/m^2^, obese $\geq$30 kg/m^2^*; CCI* Charlson Comorbidity Index | | | | | | | | | |

| Table S6. Sociodemographic characteristics and comorbidities by missing BMI status | | | |
| --- | --- | --- | --- |
|  | Non-Missing BMI | Missing BMI | P-value^1^ |
| Colon Cancer | N=3137 | N=629 |  |
| **Sex** |  |  |  |
| Female | 1575 (50.19) | 336 (53.41) | 0.142 |
| Male | 1562 (49.81) | 293 (46.59) |  |
|  |  |  |  |
| **Age** |  |  |  |
| <45 | 66 (2.10) | 19 (3.02) | <0.001 |
| 45-54 | 195 (6.21) | 59 (9.38) |  |
| 55-64 | 424 (13.52) | 115 (18.28) |  |
| 65-74 | 795 (25.35) | 82 (13.04) |  |
| 75-84 | 1137 (36.26) | 78 (12.41) |  |
| 85+ | 520 (16.57) | 110 (17.49) |  |
|  |  |  |  |
| **Deprivation Quintile** |  |  |  |
| 1 (lowest) | 761 (24.27) | 185 (29.40) | <0.001 |
| 2 | 699 (22.29) | 172 (27.34) |  |
| 3 | 677 (21.59) | 128 (20.35) |  |
| 4 | 568 (18.11) | 88 (13.99) |  |
| 5 (highest) | 432 (13.77) | 56 (8.90) |  |
|  |  |  |  |
| **Comorbidity** |  |  |  |
| CCI |  |  |  |
| 0 | 1512 (48.20) | 445 (70.73) | <0.001 |
| 1 | 763 (24.33) | 112 (17.81) |  |
| 2 | 380 (12.12) | 36 (5.73) |  |
| 3+ | 482 (15.37) | 36 (5.73) |  |
|  |  |  |  |
| CVD | 651 (20.75) | 79 (12.55) | <0.001 |
| COPD | 632 (20.15) | 52 (8.27) | <0.001 |
| Diabetes | 587 (18.71) | 17 (2.70) | <0.001 |
|  |  |  |  |
| Rectal Cancer | N=1453 | N=352 |  |
| **Sex** |  |  |  |
| Female | 574 (39.48) | 140 (39.77) | 0.926 |
| Male | 879 (60.52) | 212 (60.23) |  |
|  |  |  |  |
| **Age** |  |  |  |
| <45 | 38 (2.62) | 13 (3.69) | 0.001 |
| 45-54 | 131 (9.02) | 46 (13.07) |  |
| 55-64 | 273 (18.79) | 90 (25.57) |  |
| 65-74 | 413 (28.43) | 82 (23.30) |  |
| 75-84 | 428 (29.45) | 78 (22.16) |  |
| 85+ | 170 (11.70) | 43 (12.22) |  |
|  |  |  |  |
| 1 (lowest) | 322 (22.16) | 99 (28.13) | 0.083 |
| 2 | 309 (21.26) | 82 (23.30) |  |
| 3 | 330 (22.71) | 70 (19.89) |  |
| 4 | 261 (17.96) | 55 (15.63) |  |
| 5 (highest) | 231 (15.91) | 46 (13.07) |  |
|  |  |  |  |
| **Comorbidity** |  |  |  |
| CCI |  |  |  |
| 0 | 843 (58.04) | 286 (81.25) | <0.001 |
| 1 | 314 (21.61) | 38 (10.80) |  |
| 2 | 136 (9.36) | 15 (4.26) |  |
| 3+ | 160 (11.01) | 13 (3.69) |  |
|  |  |  |  |
| CVD | 221 (15.21) | 29 (8.24) | <0.001 |
| COPD | 235 (16.17) | 21 (5.97) | <0.001 |
| Diabetes | 230 (15.82) | 5 (1.42) | <0.001 |
| ^1^ Chi^2^ p-value  *BMI* Body Mass index, *CCI* Charlson Comorbidity Index; *CVD* cardiovascular disease (myocardial infarction, congestive heart failure, peripheral vascular disease, or cerebrovascular disease); *COPD* chronic obstructive pulmonary disease | | | |
|  |  |  |  |
|  |  |  |  |
|  |  |  |  |
|  |  |  |  |

| Table S7: Symptomatic presentation, lower GI endoscopy use, route to diagnosis and stage at diagnosis by missing BMI status | | | |
| --- | --- | --- | --- |
|  | Non-Missing BMI | Missing BMI | P-value^1^ |
| **Colon Cancer** |  |  |  |
| **New onset Symptomatic Presentation** | |  |  |
| CIBH/RB | 724 (23.07) | 136 (21.62) | 0.147 |
| Anaemia | 1061 (33.83) | 194 (30.84) |  |
| Non Red-Flag | 992 (31.62) | 228 (36.25) |  |
| Chronic only | 360 (11.48) | 71 (11.29) |  |
|  |  |  |  |
| **Had Lower GI Endoscopy after first CRC relevant symptomatic presentation** | |  |  |
| Yes | 2117 (67.47) | 383 (60.89) | 0.001 |
| No | 1020 (32.53) | 246 (39.11) |  |
|  |  |  |  |
| **Route to Diagnosis** | |  |  |
| Emergency | 875 (27.90) | 215 (34.18) | <0.001 |
| Fast-Track | 981 (31.27) | 195 (31.00) |  |
| GP Referral | 801 (25.53) | 121 (19.24) |  |
| Other In/Out | 374 (11.92) | 48 (7.63) |  |
| Screening | 106 (3.38) | 50 (7.95) |  |
| **TNM Stage at Diagnosis^2^** | |  |  |
| I | 277 (8.83) | 58 (11.86) | 0.912 |
| II | 781 (24.91) | 145 (29.65) |  |
| III | 681 (21.71) | 140 (28.63) |  |
| IV | 717 (22.87) | 146 (29.86) |  |
| Missing^3^ | 681 (21.69) | 140 (22.26) |  |
|  |  |  |  |
| **Rectal Cancer** | Non-Missing BMI | Missing BMI | P-value^1^ |
|  |  |  |  |
| **New Onset Symptomatic Presentation** | |  |  |
| CIBH/RB | 1122 (62.16) | 218 (61.93) | 0.448 |
| Anaemia | 213 (11.80) | 48 (13.64) |  |
| Non Red-Flag | 348 (19.28) | 58 (16.48) |  |
| Chronic only | 122 (6.76) | 28 (7.95) |  |
|  |  |  |  |
| **Had Lower GI Endoscopy after first CRC relevant symptomatic presentation** | |  |  |
| Yes | 1543 (85.48) | 303 (86.08) | 0.771 |
| No | 262 (14.52) | 49 (13.92) |  |
| **Route to Diagnosis** | |  |  |
| Emergency | 209 (11.58) | 42 (11.93) | 0.578 |
| Fast-Track | 817 (45.26) | 163 (46.31) |  |
| GP Referral | 532 (29.47) | 97 (27.56) |  |
| Other In/Out | 175 (9.70) | 30 (8.52) |  |
| Screening | 72 (3.99) | 20 (5.68) |  |
|  |  |  |  |
| **TNM Stage at Diagnosis^2^** | |  |  |
| I | 298 (21.83) | 59 (21.45) | 0.657 |
| II | 298 (21.83) | 52 (18.91) |  |
| III | 460 (33.70) | 99 (36.00) |  |
| IV | 309 (22.64) | 65 (23.64) |  |
| Missing^3^ | 440 (24.38) | 77 (21.88) |  |
|  |  |  |  |
| ^1^Chi^2^ p-value, ^2^Percentages for stages I to IV are for the subgroup of patients with non-missing TNM stage at diagnosis, ^3^Precentages for missing TNM stage at diagnosis refer to the total sample | | | |
| *CIBH* change in bowel habit; *RB* rectal bleeding | | | |
