# Supplementary material for: Variations in symptoms, endoscopy use and emergency diagnosis of colorectal cancer by body mass index: a retrospective cohort study using linked electronic health records in England
Source: BMJ Open. 2026 May 7;16(5):e107468. doi: 10.1136/bmjopen-2025-107468 (PMC13157786; doi:10.1136/bmjopen-2025-107468)
Supplement: Supplementary file 2 [file bmjopen-2025-107468.reviewer_comments.pdf]

PEER REVIEW HISTORY

BMJ Open publishes all reviews undertaken for accepted manuscripts. Reviewers are asked to complete a checklist review form and are provided with free text boxes to elaborate on their assessment. These free text comments are reproduced below.

ARTICLE DETAILS

Title (Provisional)

Variations in symptoms, endoscopy use and emergency diagnosis of colorectal cancer by Body Mass Index: A retrospective cohort study using linked electronic health records in England

Authors

Kurland, Jessica Sarah; Kaushal, Aradhna; Benitez Majano, Sara; Lyratzopoulos, Georgios; Renzi, Cristina

---

VERSION 1 - REVIEW

**Reviewer**	**1**
**Name**	**Boakye, Daniel**
**Affiliation**	**University of the West of Scotland**
**Date**	**07-Aug-2025**
**COI**	**None**

Kurland and colleagues employed a retrospective cohort design to evaluate variations in symptoms, use of endoscopy, and emergency diagnosis of CRC by BMI in the UK. The findings are expected and relevant, and the manuscript is nicely written.

Below are some statistical considerations to further strengthen the methodology and reporting of the findings of the manuscript.

1. The authors should specify that the design is “retrospective cohort” throughout the manuscript to avoid misinterpretation as a prospective cohort design.

2. CRC screening is usually not recommended for those younger than 50 years in the UK. As such, it would be appropriate for the authors to include only CRC cases diagnosed at 50+ years in the analysis of BMI and endoscopic use. Else, the results are not interpretable.

3. Close to 20% of the eligible patient population were excluded because of missing data on BMI. Were they comparable to the included patient population in terms of age, sex, deprivation, symptoms, presentation, endoscopic use? The authors discussed this as a potential study limitation in the discussion section, but it is also essential to create another

table comparing the included and excluded sample (missing BMI) according to the variables listed in Table 1.

4. The current Table 1 is confusing, as the authors analyzed missing BMI, including the Chi-square test. The authors named the first column as “All patients,” but those with missing BMI were excluded from the analysis according to Figure 1.

5. The authors should carefully check the numbers for consistency. Figure 1 shows that colon cancer cases included in the analysis were 1729. However, Tables 1, 2, and S1 show different numbers. Same for rectal cancer.

6. There are few patients in the underweight group, especially ages <65. Can the authors regroup age using these categories to improve precision of the estimates (<55, 55-64, 65-74, 75-84, 85+)?

7. Can the authors provide a rationale for the cut-off used for the categorization of number of visits?

**Reviewer**	**2**
**Name**	**Huei, Tan**
**Affiliation**	**Hospital Sultanah Aminah**
**Date**	**13-Oct-2025**
**COI**	**None**

---

it is a good effort from the authors. however, with the AI software nowadays, it is easier to write this type of paper.

this paper found an expected link of overweight or obesity that has association with colorectal cancer in general.

the results solely are postulation / association which add no new finding to the current literature.

extensive statistical analysis with complex data presentation lacking diagram or figure result in poor readability value for many busy clinician.

VERSION 1 - AUTHOR RESPONSE

Reviewer 1: Dr Daniel Boakye

Responses to Reviewer 1’s comments<br><br>We would like to thank Reviewer 1 for their thoughtful and helpful comments. Please find our responses to each comment in the table below.	
Comment	Response

1. The authors should specify that the design is “retrospective cohort” throughout the manuscript to avoid misinterpretation as a prospective cohort design.	We agree and have specified the study design within the title and throughout the manuscript
2. CRC screening is usually not recommended for those younger than 50 years in the UK. As such, it would be appropriate for the authors to include only CRC cases diagnosed at 50+ years in the analysis of BMI and endoscopic use. Else, the results are not interpretable.	We thank the reviewer for this comment. On a technical note, during our study period of 2011-2015, the minimum age for screening invitation for colorectal cancer had been the age of 60, not 50 (although this age threshold has been revised downwards since 2021 onwards).<br><br>However, the broader comment is about whether restricting the analysis to patient groups above the screenable age threshold (for England) would be appropriate. Implicitly this suggestion assumes that endoscopies are chiefly generated by screening participation, but in truth, at least in England, the great majority of colonoscopies relate to the diagnostic management of patients presenting to primary care with symptoms. Furthermore, and perhaps more crucially, our study a priori and solely focused on symptomatic patients who had presented to the GP with relevant CRC symptoms. Of course, age may impact GP decision making regarding referrals for endoscopy, therefore we have included age as a variable in the adjusted regression analyses to account for this.<br><br>Therefore, including symptomatic patients across the entire age spectrum within the sample is the most appropriate study population given the study aims and design.
3. Close to 20% of the eligible patient population were excluded because of missing data on BMI. Were they comparable to the included patient population in terms of age, sex, deprivation, symptoms, presentation, endoscopic use? The authors discussed this as a potential study limitation in the discussion section, but it is also essential to create another table comparing the included and excluded sample (missing BMI) according to the variables listed in Table 1.	In response to comments 3 and 4, we have amended tables 1 and 2, and added two additional tables to the appendix (Tables S6 and S7).<br><br>Tables 1 and 2 now show the descriptive characteristics of only the analytical sample included in the regression analyses (i.e. patients with available BMI data).<br><br>Supplementary tables 6 and 7 contain a comparison of descriptive characteristics (sociodemographic, comorbidity, symptoms and route to diagnosis) across those with

	missing and non-missing BMI data and a description of this has been added to the results section (see Sensitivity Analysis section).<br><br>Figure 1 has also been amended to reflect this change.
4. The current Table 1 is confusing, as the authors analysed missing BMI, including the Chi-square test. The authors named the first column as "All patients," but those with missing BMI were excluded from the analysis according to Figure 1.	See response for comment 3 above.
5. The authors should carefully check the numbers for consistency. Figure 1 shows that colon cancer cases included in the analysis were 1729. However, Tables 1, 2, and S1 show different numbers. Same for rectal cancer.	Thank you for flagging this. The numbers have been checked and amended. All tables and figures now show the correct sample size for the descriptive and regression analysis. For the descriptive analysis this is: colon cancer N=3137, rectal cancer N=1453. For the main analysis this is: colon cancer N=3031, rectal cancer N=1401. The colon cancer N=1729 refers to a subgroup analysis of patients with new onset red flag symptoms, and this figure is consistent across relevant figures and tables (Figure 2a, Table S3).
6. There are few patients in the underweight group, especially ages <65. Can the authors regroup age using these categories to improve precision of the estimates (<55, 55-64, 65-74, 75-84, 85+)?	There are fewer than 80 patients in the underweight category across the age spectrum in our sample. Therefore, unfortunately, even with recategorizing the age categories to include a greater number of patients with underweight at younger ages, the sample sizes would remain too small to draw any meaningful conclusions about this group. We have acknowledged this in the limitation section.
7. Can the authors provide a rationale for the cut-off used for the categorization of number of visits?	Number of GP visits was included as a continuous count variable – there were no cut-off values as such. We did use a time cut off, only including primary care visits within 12 months pre-diagnosis, and excluding any recorded within the 30 days pre-diagnosis. Visits within 30 days pre-diagnosis were excluded as customary in this research field, as the 30 days pre-diagnosis are integral to the final stages of the diagnostic process, which would therefore not represent new or missed opportunities for diagnosis. This additional detail has been added to the

	manuscript – please see 3rd paragraph of the Study Variables section within the Methods.

Reviewer 2: Dr Tan Huei

Review 2 Comments: it is a good effort from the authors. however, with the AI software nowadays, it is easier to write this type of paper.
this paper found an expected link of overweight or obesity that has association with colorectal cancer in general.
the results solely are postulation / association which add no new finding to the current literature.
extensive statistical analysis with complex data presentation lacking diagram or figure result in poor readability value for many busy clinician.

Response to reviewer 2's comments: We appreciate it is not possible to assure the quality of the peer-review process, but we feel that this is a very short (77-word) and superficial review that does not support or enhance the editorial and peer-review process. The Reviewer acknowledges 'good effort' by our team, but only to then pejoratively imply that we have used AI software to write the paper. Their 3rd sentence seems to indicate that they feel that our paper is about examining the association between obesity and colorectal cancer risk – which is not our research question, rather we have studied whether obesity is associated with presenting symptoms of colorectal cancer, endoscopy use, and risk of diagnosis as an emergency, representing entirely different research questions to the one hypothesised by the Reviewer. This represents a surprising and fundamental misunderstanding of the study's purpose by the Reviewer. The Reviewer indicates that the findings do not add to current literature, but we believe they refer to literature about associations between obesity and the risk of developing colorectal cancer (as above) – not literature about our study's own research questions. We cover in Discussion how the findings relate to prior relevant literature and how they enhance the evidence base. In conclusion, we do not feel the comments were valid or detailed enough for us to be able to make any changes to improve the paper.

VERSION 2 - REVIEW

**Reviewer**	**1**
**Name**	**Boakye, Daniel**
**Affiliation**	**University of the West of Scotland**
**Date**	**30-Jan-2026**
**COI**	

Kurland and colleagues employed a retrospective cohort design to assess variations in symptoms, endoscopy use, and emergency diagnosis of CRC according to BMI in England. The methods are appropriate, and the findings are interesting.

Incorporating the suggestions below would further enhance the content of the manuscript.

Introduction

- Can the authors summarize the findings of the previous studies on BMI and its associations with EP and CRC stage at diagnosis mentioned in the introduction section?

Methods

- What was the definition of CVD in Table 1 and the other tables in the manuscript? Can the authors clarify?

- Can the authors also analyze CVD, COPD, diabetes as explanatory variables in the association with EP and fast-track referrals (e.g., Figure 2, Table S2, Table S3)? For those analyses, they should remove CCI score from the model.

General

- Manuscript needs to be proofread

VERSION 2 - AUTHOR RESPONSE

We would like to thank Dr Boakye for reviewing our paper and offering helpful comments. Please find our responses below.

Introduction
Comment: Can the authors summarize the findings of the previous studies on BMI and its associations with EP and CRC stage at diagnosis mentioned in the introduction section?

Response: A summary of the findings has been added to the introduction (see Introduction paragraph 2)

Methods
Comment: What was the definition of CVD in Table 1 and the other tables in the manuscript? Can the authors clarify?

Response: The presence of CVD is defined as at least one of the following conditions: myocardial infarction, congestive heart failure, peripheral vascular disease, or cerebrovascular disease. This detail has been added to the methods section and footnotes of relevant tables.

Comment: Can the authors also analyze CVD, COPD, diabetes as explanatory variables in the association with EP and fast-track referrals (e.g., Figure 2, Table S2, Table S3)? For those analyses, they should remove CCI score from the model.

Response: Thank you for this comment. The requested analyses have been included for emergency presentations (see Supplementary Table 4) and show little difference in the results when compared to the model using CCI instead of individual comorbidities. We also investigated the possibility of running these sensitivity analyses for our second outcome (fast-track referrals), but as this was conducted on a subset of patients, these models would have been statistically underpowered to due to the reduced number of patients and small numbers with these individual comorbidities. Given that there was no change in the results for EP, we think it is also unlikely there would be a difference when comparing results for fast-track referrals between models using CCI or individual comorbidities.

General

Comment: Manuscript needs to be proofread

Response: Thank you for flagging this. The manuscript has been checked and errors have been amended.
